# Injection of Aquafilling^®^ for Breast Augmentation Causes Inflammatory Responses Independent of Visible Symptoms

**DOI:** 10.1007/s00266-020-01949-y

**Published:** 2020-09-16

**Authors:** Michał Chalcarz, Jakub Żurawski

**Affiliations:** 1 Chalcarz Clinic, Aesthetic Surgery, Aesthetic Medicine, Poznań, Poland; 2 Bieńkowski Medical Center - Plastic Surgery, Bydgoszcz, Poland; 3 Department of Immunobiology, Poznan University of Medical Sciences, Poznań, Poland

**Keywords:** Aquafilling^®^, Polyamide, Polyacrylamide, Breast augmentation, Inflammation, Complications, Surgical treatment

## Abstract

**Background:**

A major concern related to modern surgery is to evaluate and address the complications associated with breast enlargement using Aquafilling^®^ injection. This study aimed to assess the effect of Aquafilling^®^ injection on immune response in such patients.

**Methods:**

For four patients who consulted a surgeon after receiving Aquafilling^®^ injection, medical history of the patients was taken; based on imaging examinations, Aquafilling^®^ was removed. Samples were processed for histopathological and immunohistochemical examination. For detecting tissue antigens in histopathological samples, monoclonal antibodies against CD3 (lymphocytes T), CD 20 (lymphocytes B), and CD68 (macrophages) were used. By analyzing the images, the number of immune cells (lymphocytes T, lymphocytes B, and macrophages) and immunohistochemical reaction area were semiquantitatively evaluated.

**Results:**

Different clinical features were observed in each patient after receiving Aquafilling^®^ injection. In samples obtained from four patients, lymphocytes T (CD3), lymphocytes B (CD20), and macrophages (CD68) tissue expressions were observed. Statistically significant variations in the number of lymphocytes B (CD20) and macrophages (CD68), and differentiation of immunohistochemical reaction area for lymphocytes T (CD3) and lymphocytes B (CD20) were observed.

**Conclusions:**

Inflammation is elevated in patients who received Aquafilling^®^ injection. Medical imaging should be carried out in all such patients even if there are no visible symptoms. Removal of Aquafilling^®^ can reduce the inflammation and risk of neoplastic progression in the patients. The influence of time elapsed since Aquafilling^®^ injection and intensity of immune response requires further validation.

**Level of Evidence IV:**

This journal requires that authors assign a level of evidence to each article. For a full description of these Evidence-Based Medicine ratings, please refer to the Table of Contents or the online Instructions to Authors www.springer.com/00266.

## Introduction

Aquafilling^®^, produced by BIOTRH s. r. o., Prague, Czech Republic, was introduced in 2005 as a soft tissue filler to model face and buttocks, and subsequently, for augmentation of breasts. It is a hydrophilic gel and is composed of 98% physiological saline and 2% polyamide [1–4]. However, according to the Korean Food and Drug Administration (KFDA), its composition is 98% physiological saline and 2% polyacrylamide [5]. Aquafilling^®^ has been used in the European Union, Malaysia, South Korea, Serbia, and Turkey [6]. However, the United States Food and Drugs Administration (USFDA) has not approved its use as an injectable filler for breast augmentation [5].

Aquafilling^®^ was created to overcome the harmful effects of polyacrylamide-based fillers; unfortunately, its use is linked with various health concerns such as mastalgia, breast deformation, and inflammation of mammary glands in patients, as indicated by test results carried out in South Korea [4, 7] and Turkey [8]. It has also been associated with difficulties in breastfeeding, and migration of filler to the cervical section, the wall of the chest, the abdominal cavity, armpits, pelvis, and labia. Moreover, it has been reported to cause inflammation, abscess, and fistula of mammary glands [1, 4, 6, 7]. However, no study has yet reported on the adverse effects caused by Aquafilling^®^ on the immune system, or investigated whether these changes depend on the visible symptoms and ailments observed in patients, the amount of filler used, or the time lapse from its injection.

## Objectives

This research aimed to compare and evaluate unfavorable symptoms observed in women after receiving Aquafilling^®^ injection in both breasts, as well as changes in histopathological and immunohistochemical parameters of the tissues obtained during filler’s surgical removal.

## Materials and Methods

### Clinical Data

The study included four female patients who consulted a surgeon after receiving Aquafilling^®^ injection. Data of the examined patients are presented in Table 1 for comprehension (Table 1).

**Table 1:** Data of the examined patients

Parameter	Patient 1	Patient 2	Patient 3	Patient 4
Volume of injected Aquafilling^®^ into breasts (ml)	100 ml in each breast	200 ml in each breast	230 ml in left breast 260 ml in right breast	105 ml in each breast
Time elapsed since noticing first side effects after filler injection (months)	Did not occur^a^	1 month	3 months	20 months
Type of unfavorable symptoms	Did not occur^a^	Migration of Aquafilling^®^ below the inframammary fold	Pain in the right breast during physical effort, while raising hands, driving the car, or lying on the side; deformation of both breasts	Pain in both breasts preceding each menstruation cycle; deformation of the right breast in the upper pole
Breast medical imaging performed before surgical removal	Ultrasonography (USG)	Ultrasonography (USG) and magnetic resonance imaging (MRI)	Ultrasonography (USG)	Ultrasonography (USG)
Time elapsed since Aquafilling^®^ injection to its removal procedure	36 months after injection	28 months after injection	12 months after injection	37 months after injection
Morphological parameters: leukocyte, erythrocyte, monocyte counts, hemoglobin level	In the reference range	In the reference range	In the reference range	In the reference range
Biochemical parameters: C-reactive protein (CRP), urea, creatinine level, activated partial thromboplastin time (APTT) international normalized ratio (INR)	In the reference range	In the reference range	In the reference range	In the reference range
Aquafilling^®^ presence in the pectoral muscles	Yes	Yes	Yes	Yes

^a^She visited the clinic because the filler was still visible in an ultrasound scan despite a three-year lapse, and because her friends who underwent the same procedure had suffered from complications

Patient 1 was injected with 100 ml of Aquafilling^®^ in each breast for augmentation. This patient did not complain of any symptoms or ailments following filler injection. However, since the filler was still visible in an ultrasound scan despite a three-year lapse, and because she was aware of the profoundly serious complications observed in other patients, she visited the clinic. She underwent removal of Aquafilling^®^ 36 months after injection. The patient did not provide consent to photography.

**Fig. 1:**
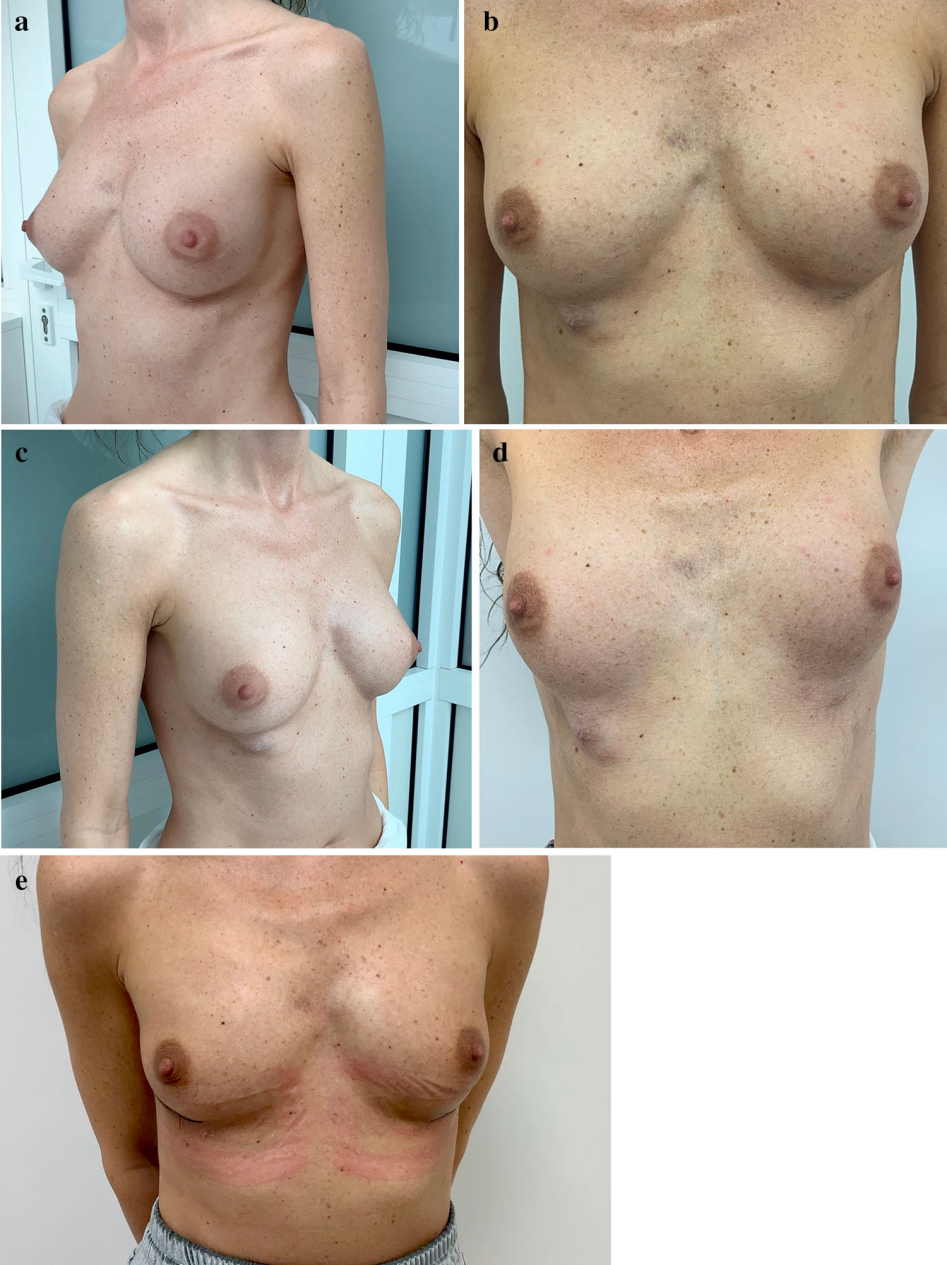
Photographs of patient 2 **a**–**c** preoperatively in the standing position: visible displacement of filler below the inframammary fold of both breasts, and **d** with raised hands and **e** on postoperative 14 day showing no complications

Patient 2 was injected with 200 ml of Aquafilling^®^ in each breast for breast augmentation. After one month, some part of the filler was displaced in the right breast, creating a vessel below the inframammary fold. Approximately 27 months later, a similar change was observed in the left breast. Breast ultrasonography and magnetic resonance imaging (MRI) were performed, and Aquafilling^®^ was removed 28 months after injection (Fig. 1).

**Fig. 2:**
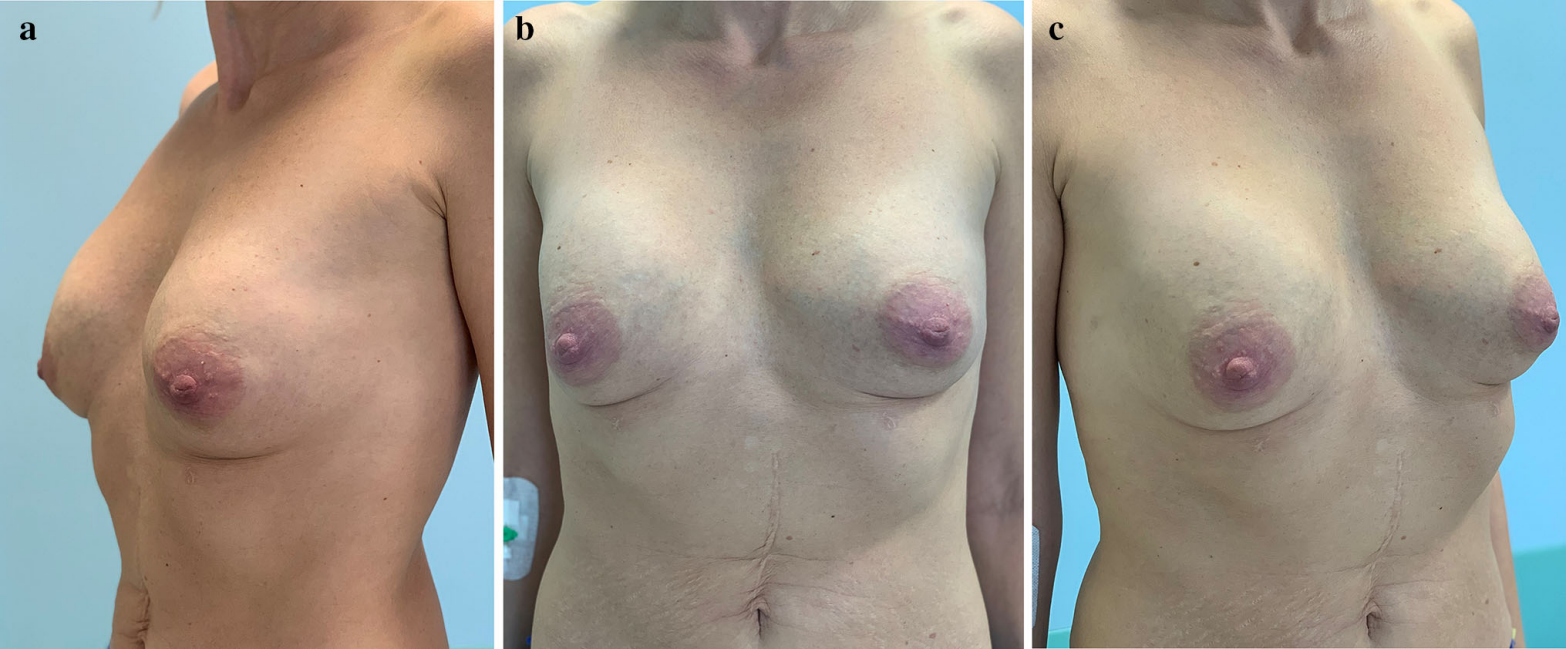
Photographs of patient 3 **a**–**c** preoperatively in the standing position: visible deformation of both breasts

Patient 3 was injected with 230 and 260 ml (initially 230 ml and another 30 ml 2 weeks later) of Aquafilling^®^ in the left and right breasts, respectively. Three months after the first injection, the patient experienced pain in the right breast during physical effort, while raising hands, driving the car, or lying on the side. After another two months, deformation of both breasts was observed. Breast ultrasonography was performed, and Aquafilling^®^ was removed 12 months after injection (Fig. 2).

**Fig. 3:**
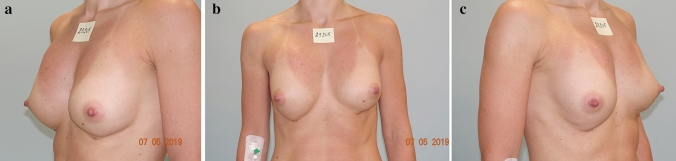
Preoperative images of patient 4 **a**–**c** in the standing position: visible deformation of the right breast in the upper pole observed after 2.5 years

Patient 4 was injected with 105 ml of Aquafilling^®^ in each breast (initially 75 ml, and another 30 ml after 9 months) to augment the breasts. Twenty months after the first injection, the patient experienced pain in both breasts preceding each menstruation cycle, lasting for about 3 days; 2.5 years after the first injection, a deformation of the right breast in the upper pole was observed. Breast ultrasonography was performed, and the Aquafilling^®^ material was surgically removed 37 months after injection (Fig. 3).

In each patient, morphological parameters such as leukocyte, erythrocyte, and monocyte counts, hemoglobin levels, as well as biochemical parameters, C-reactive protein (CRP), urea creatinine levels, activated partial thromboplastin time (APTT), and international normalized ratio (INR), were within the reference range.

**Fig. 4:**
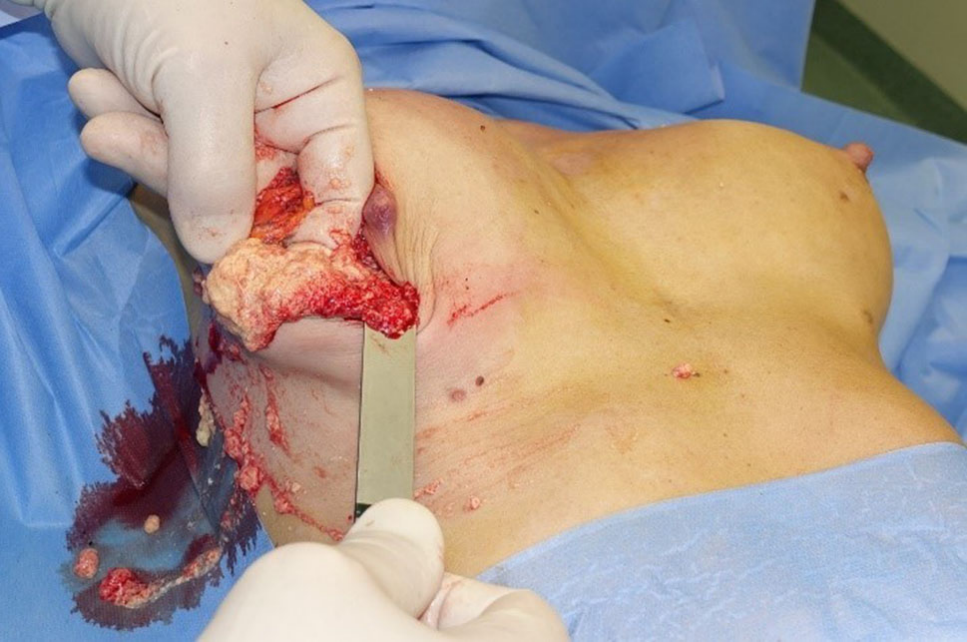
Removal of Aquafilling^®^ from breasts

**Fig. 5:**
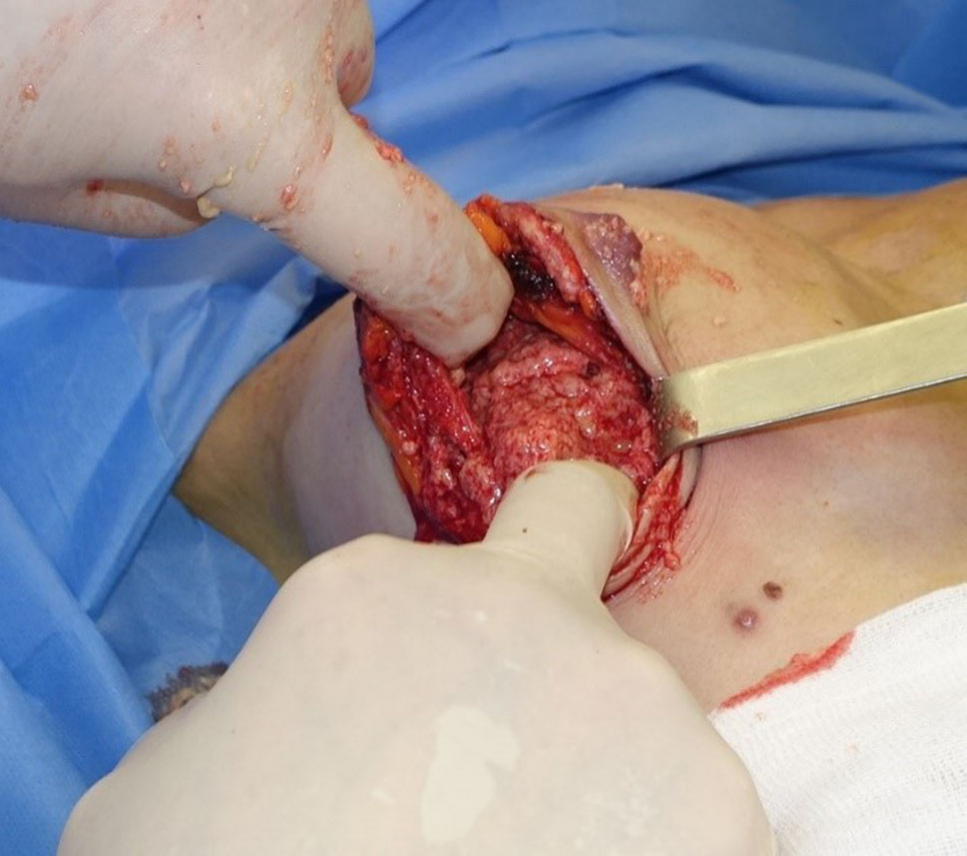
Pectoralis major muscle infiltrated with Aquafilling^®^

**Fig. 6:**
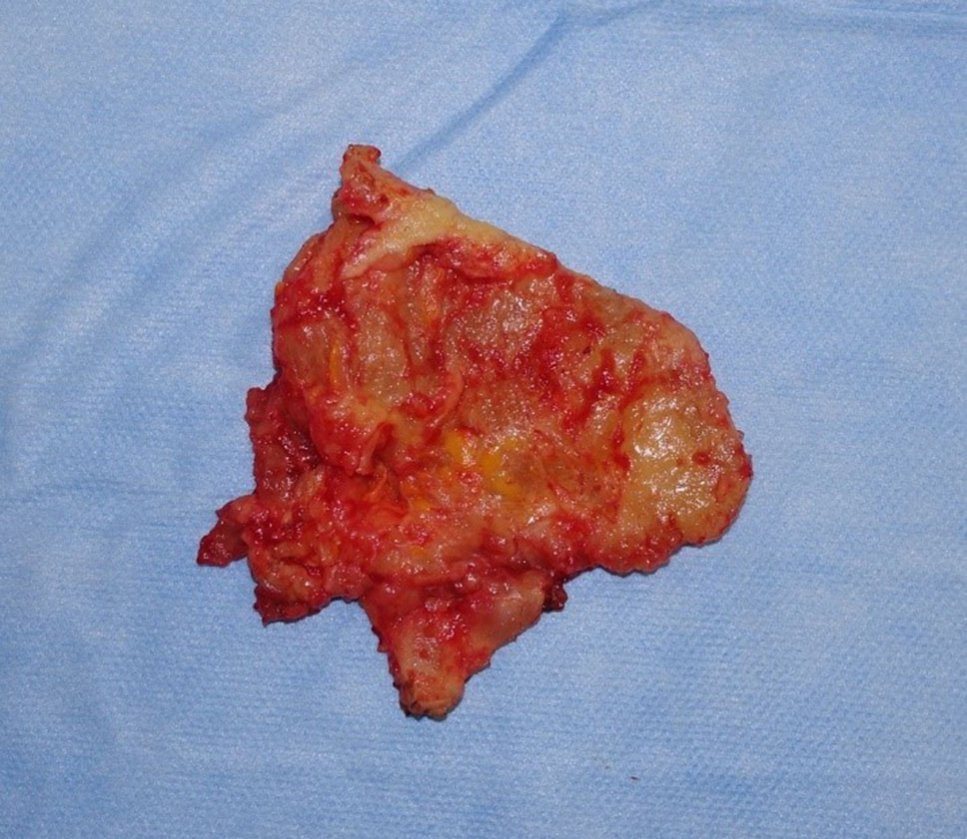
Removed section of the inflamed pectoralis major muscle infiltrated with Aquafilling^®^

Therefore, all four patients underwent surgery under general anesthesia for the removal of Aquafilling^®^ and the inflamed adjacent tissue. During the surgical removal of the filler, it was observed to be present in the pectoral muscles of each patient (Figs. 4, 5, 6), and tissue samples were obtained for histopathological examination. All patients were informed preoperatively that complete removal of the injected filler was impossible, and MRI re-examination would be necessary 6 months postoperatively.

### Histopathological and Immunohistochemical Examinations

The tissue samples were processed according to a standard histological procedure [9]. Samples for histopathological evaluation were stained with hematoxylin and eosin. For detecting tissue antigens in histopathological samples, monoclonal antibodies against CD3 (lymphocytes T), CD20 (lymphocytes B), and CD68 (macrophages) were used. Activity of endogenous peroxidase was blocked with a 3% H_2_O_2_ solution. The tissue samples were incubated at 25º C with the primary antibody. Next, the samples were rinsed and incubated with secondary antibodies. In all the samples 3, 3′-diaminobenzidine (DAB) chromogen was used to locate the antigen.

Immunohistochemical slides were visualized and imaged using the Olympus BX 43 light microscope (Olympus, Tokyo, Japan) and XC 30 digital camera. Ten images were captured with 100 × magnification. By analyzing the images, the number of immune cells (lymphocytes T, lymphocytes B, and macrophages), as well as immunohistochemical reaction areas, was semiquantitatively evaluated. Calculations were performed using cellSens Dimension software (Olympus) [10]. For evaluating cell number and reaction area, 60 specimens (approximately 1225 cells/specimen) for lymphocytes T (CD3); 44 specimens (approximately 918 cells/specimen) for lymphocytes B (CD20); and 60 specimens (1096 cells/specimen) for macrophages were used. The cellSens Dimension software performed phase analysis of the stained samples, involving automatic detection of objects by their color, hue intensity, or shape. For our cases, the hue criterion was chosen (brown DAB chromogen). The software automatically classified the samples based on the pre-defined threshold values. In the samples, immunopositive cells were evaluated.

### Statistical Analysis

To evaluate the differences in immunopositive cell number and immunohistochemical reaction area, statistical package Statistica 13.3 (StatSoft, Tulsa, USA) was used.

Variables were described using descriptive statistics: expected value, standard deviation, median *Q*_1_–*Q*_3_, quantile, minimum, and maximum as well as statistics associated with variable dispersion. Descriptive statistics were indicated by 95% confidence intervals.

To evaluate statistically significant differences in immunopositive cell number as well as immunohistochemical reaction area, one-way ANOVA or Kruskal–Wallis test was performed, followed by a post hoc test.

## Results

### Histopathological and Immunohistochemical Examinations

**Fig. 7:**
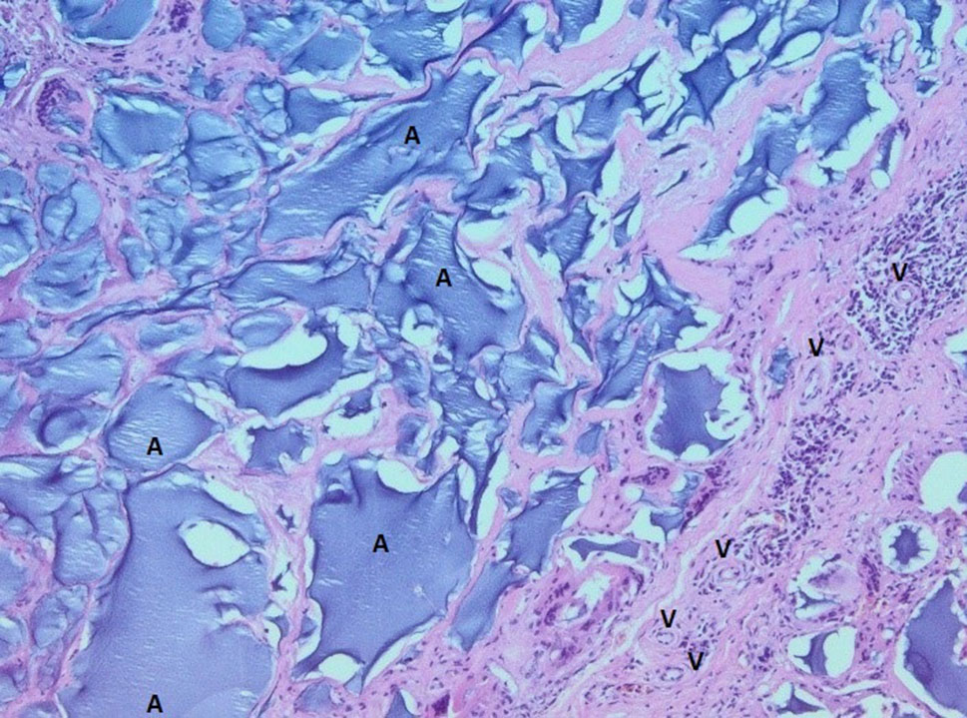
Abundant basophilic Aquafilling^®^ (*A*). Surrounded by diffuse inflammatory infiltrates. Blood vessels with thickened walls (*V*). HE stained. Magnification 50 ×

**Fig. 8:**
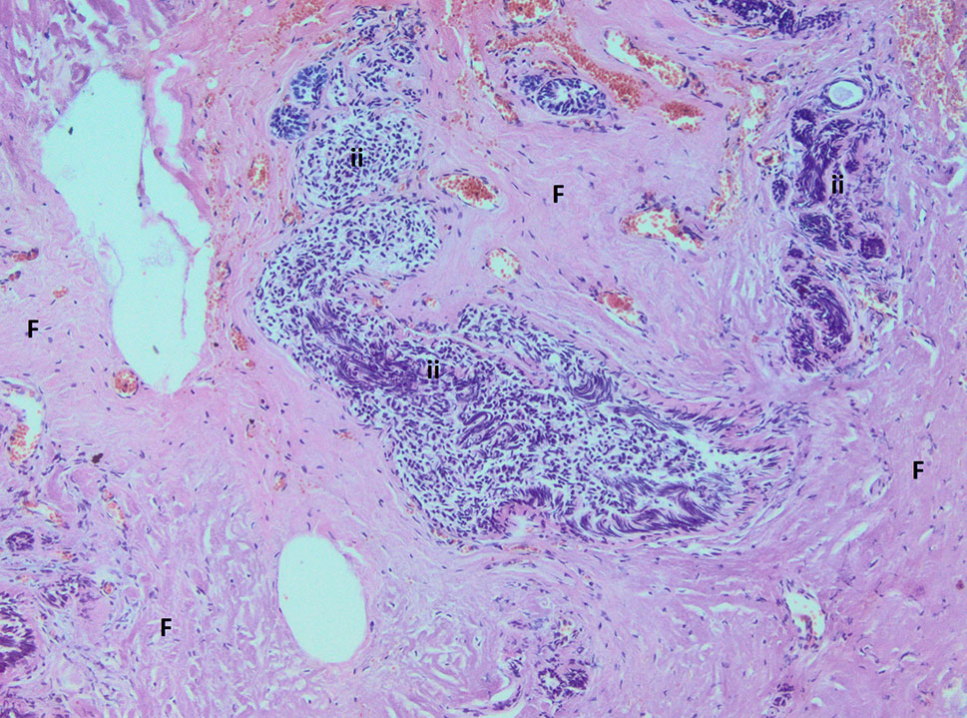
Extensive fibrosis (*F*) with inflammatory infiltrates (ii). HE stained. Magnification 50 ×

**Fig. 9:**
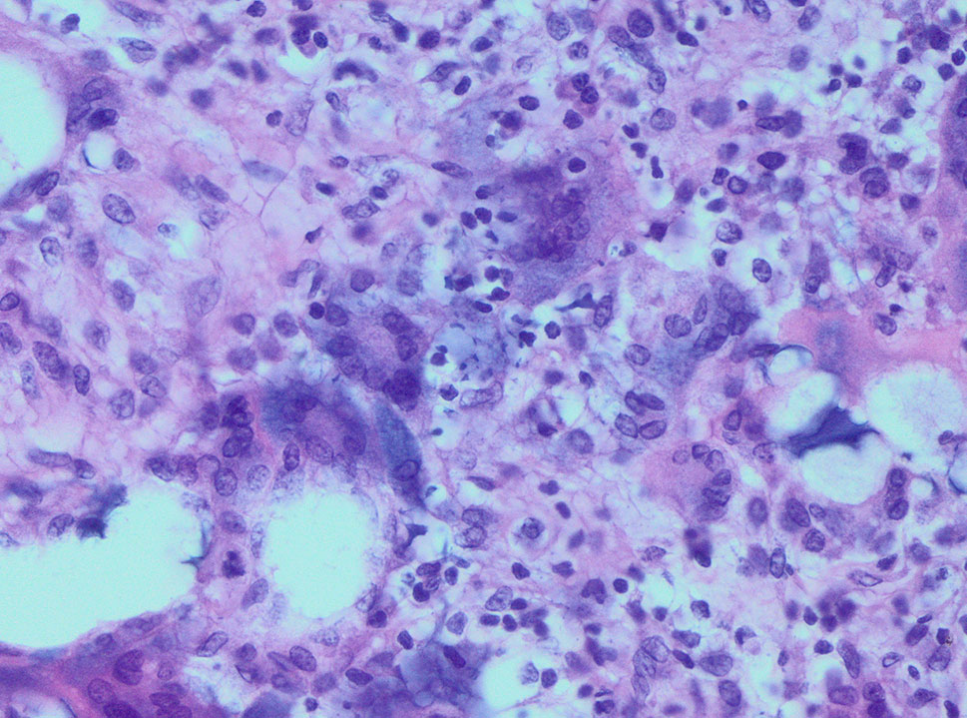
Inflammatory infiltrates with mononuclear cells. HE stained. Magnification 400 ×

**Fig. 10:**
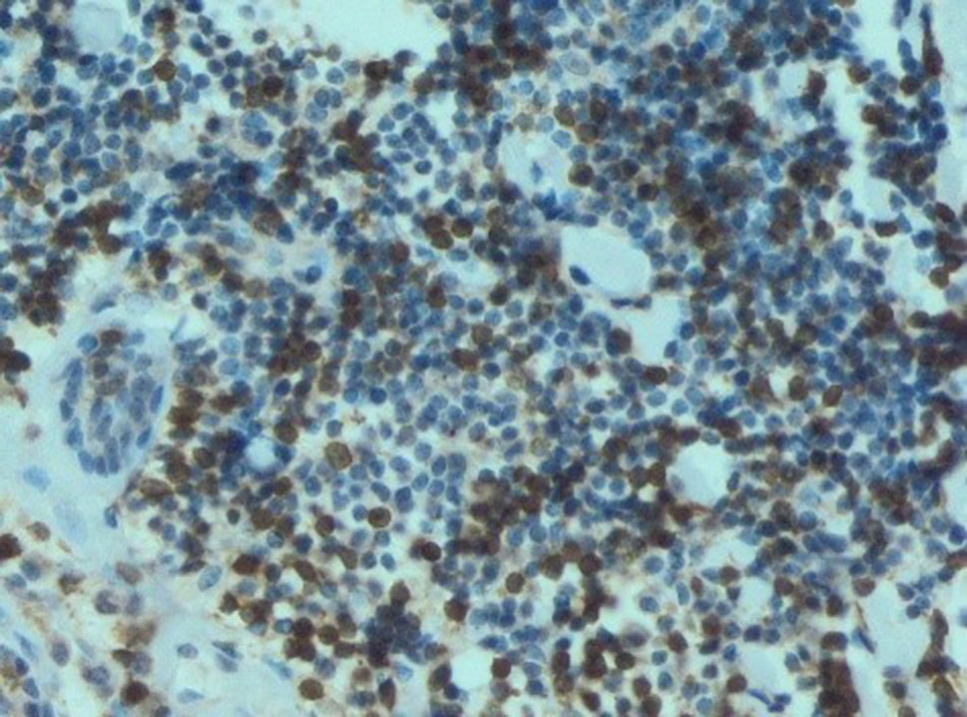
T cell (CD3) tissue expression. Immunohistochemical staining. Magnification 100 ×

**Fig. 11:**
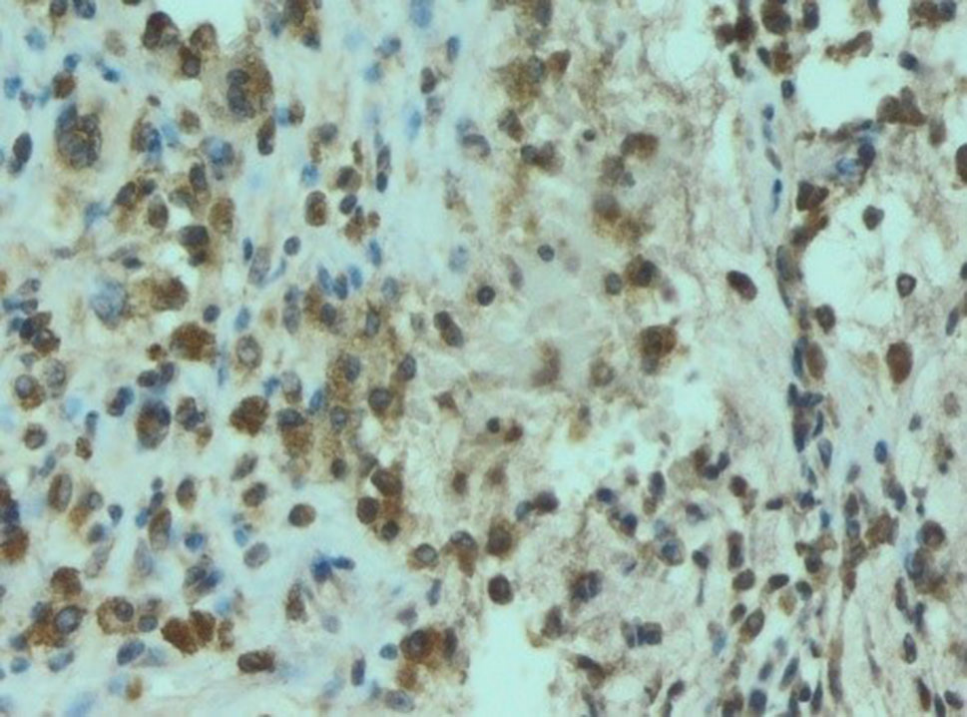
Macrophages (CD68) tissue expression. Immunohistochemical staining. Magnification 100 ×

**Fig. 12:**
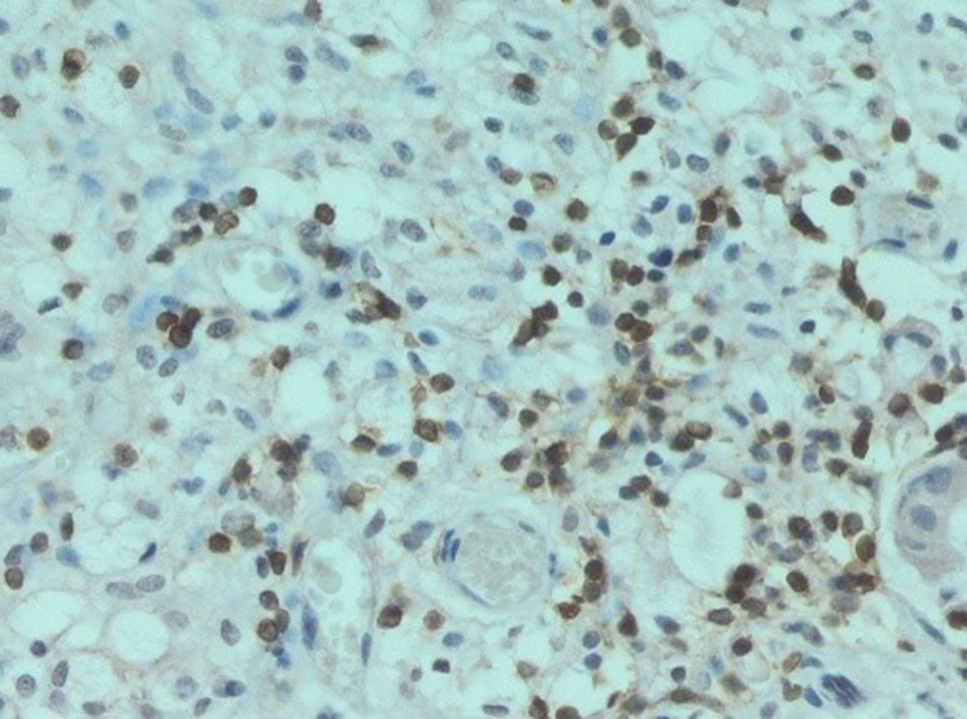
B cell (CD20) tissue expression. Immunohistochemical staining. Magnification 100 ×

In all examined samples obtained from four patients, similar changes were observed in the mammary gland. Fibrous connective tissue was found partly hyalinized with foci of fatty tissue. Within it, abundant basophilic, homogenous content of Aquafilling^®^ with extensive infiltration of mononuclear cells and numerous small blood vessels were observed. Some of the blood vessels had thickened walls. In others, endothelium layers were separated (Figs. 7, 8, 9). Furthermore, the expression of lymphocytes T (CD3), lymphocytes B (CD20), and macrophages (CD68) was also observed in the tissue samples (Figs. 10, 11, 12).

A significant difference was observed in the number of lymphocytes B (CD20) cells and macrophages (CD68) (Table 2). Variations in the cell number of lymphocytes B (CD20) were noted at a level of *p* = 0.0015 and were associated with statistically significant difference between lymphocytes number in Patient 1 and 2 on a level of *p* = 0.0005. In contrast, a significant variation in the macrophage number (CD68) was noted at a level of *p* = 0.0002 as a result of statistically significant difference in the macrophages number between Patients 2 and 3 at a level of *p* = 0.0001. Despite the lack of statistically significant difference in the lymphocytes T (CD3) number, in patient 3, lymphocytes T (CD3) number was the highest.

**Table 2:** Immunopositive cell number in examined patients

Patient number	Total cell number	Expected value ± SD	Median (*Q*_1_/*Q*_3_)	Min/max	95% of average confidence	*p* value (post hoc)
*Lymphocytes T (CD3)*						
Patient 1	13,264	1326.4 ± 905.6	1076 (821/3140)	238/3140	678.5/1974.3	0.2085
Patient 2	8488	2182.2 ± 1804.8	1510 (460/4908)	306/4908	1337.5/3026.8	
Patient 3	43,643	662.1 ± 101.5	660.5 (594.5/815)	427/815	614.5/709.6	
Patient 4	13,241	848.8 ± 271	850 (661/1189)	430/1189	654.9/1042.7	
In total	78,636	1310.6 ± 1274.7	745 (585/4908)	238/4908	981.3/1639.9	
*Lymphocyte B (CD20)*						
Patient 1	2462	223.8 ± 207.2	115 (89/694)	64/694	84.6/363	0.0015 1/2
Patient 2	8790	1469.8 ± 1316.4	678 (404/3421)	178/3421	835.3/2104.4	
Patient 3	27,927	485.3 ± 330.7	361.5 (272/968)	250/968	0/1011.5	
Patient 4	1941	879 ± 999.2	347 (197/3026)	144/3026	164.2/1593.8	
In total	41,120	934.5 ± 1107.9	416.5 (190/3421)	64/3421	597.7/1271.4	
*Macrophages (CD68)*						
Patient 1	10,496	1049.6 ± 259.7	1038.5 (845/1439)	687/1439	863.9/1235.4	0.0002 2/3
Patient 2	10,807	990.9 ± 1031.4	631.5 (481.5/4269)	324/4269	508.1/1473.6	
Patient 3	19,817	1435.7 ± 360.8	1532.5 (1154/1925)	626/1925	1266.8/1604.5	
Patient 4	28,713	1080.7 ± 224.8	1161 (907/1357)	658/1357	919.9/1241.5	
In total	69,833	1163.9 ± 664.1	1097.5 (701/4269)	324/4269	992.3/1335.5	

As shown in Table 3, a significant difference was observed in immunohistochemical reaction areas for lymphocytes T (CD3) as well as lymphocyte B (CD20). Statistically significant variations were observed in the immunohistochemical reaction area for lymphocytes T (CD3) among the patients (*p* = 0.0003). This variation was associated with detailed differences between immunohistochemical reaction area calculated for Patient 1 and those calculated for other patients at the following levels: *p* = 0.0056, *p* = 0.0004, and *p* = 0.0019. Immunohistochemical reaction area calculated for lymphocytes B (CD20) also varied significantly among examined patients at a level of *p* = 0.0001. This differentiation was associated with statistically significant differences observed between the immunohistochemical reaction area calculated for Patient 1 and those calculated for Patients 2 and 3 (*p* = 0.0001 vs. *p* = 0.0123), between Patients 2 and 4 (*p* = 0.0019), and between Patients 3 and 4 (*p* = 0.0481).

**Table 3:** Immunohistochemical reaction area in examined patients (µm^2^)

Patient number	Total area	Expected value ± SD	Median (*Q*_1_/*Q*_3_)	Min/max	95% of average confidence	*p* value (post hoc)
*Lymphocytes T (CD3)*						
Patient 1	3027.7	3027.7 ± 3312.3	1771.2 (720.1/4263.2)	281.2/10,893.2	658.2/5397.1	0.0003 1/2; 1/3; 1/4
Patient 2	11,177.2	10,335 ± 5129.5	9039.1 (7156.1/13,613)	2710.6/25,112.3	7934.3/12,735.7	
Patient 3	10,335.0	10,942.8 ± 2603.6	10,497.9 (9481.6/13,032.7)	5823.9/15,969.8	9724.2/12,161.3	
Patient 4	10,942.8	11,177.2 ± 3960.5	11,272.2 (8406.9/14,330.8)	4986.8/17,072.1	8344/14,010.3	
In total	35,482.6	9460.1 ± 4820.8	10,026 (5959.9/13,032.7)	281.2/25,112.3	8214.7/10,705.4	
*Lymphocytes B (CD20)*						
Patient 1	501.8	501.8 ± 449.5	271.3 (232.8/897.4)	201.5/1584.9	199.9/803.8	0.0001 1/2; 1/3; 2/4; 3/4
Patient 2	790.7	16,639.9 ± 8648.2	19,233.6 (8961.2/23,068.6)	196.6/26,835.4	12,471.6/20,808.2	
Patient 3	16,639.9	16,357.9 ± 9840.3	16,629.1 (7922.8/24,793)	6468.5/25,705.1	699.8/32,016.1	
Patient 4	16,357.9	790.7 ± 728.7	407.3 (311.9/1538.3)	101/2143.3	269.4/1312	
In total	34,290.4	8977.7 ± 10,158.5	1858.5 (313.9/19,467)	101/26,835.4	5889.2/12,066.1	
*Macrophages (CD68)*						
Patient 1	17,082.2	17,082.2 ± 1685.9	16,989.9 (15,735.7/18,605)	14,393.1/19,272.3	15,876.1/18,288.2	0.2877
Patient 2	17,365.0	16,192.5 ± 7405.1	15,575.4 (12,546.7/18,237.3)	7396.3/41,091.1	12,726.8/19,658.2	
Patient 3	16,192.5	15,787.6 ± 2678.6	15,181.3 (14,234.6/16,609.8)	12,177.1/22,546.7	14,534/17,041.2	
Patient 4	15,787.6	17,365 ± 3919.7	16,545.8 (14,876/16,929.6)	14,780.4/27,627.4	14,561/20,169	
In total	66,427.3	16,401.2 ± 4808.9	15,929 (14,422.2/17,656.6)	7396.3/41,091.1	15,159/17,643.5	

## Discussion

Health concerns associated with Aquafilling^®^ injection are relatively new, as evident by the small number of reports on female patients undergoing surgery due to inflammation and other complications. Previously published researches were performed on a smaller number of female patients, i.e., one [1, 6–8], two [2], and three [3], indicating that these studies are just in their initial stages all over the world.

In the existing literature [1, 2, 4, 6–8], distinct clinical features have been reported in patients who underwent breast augmentation. Jung et al. [1] described patient characteristics as repeated wound dehiscence and fluid discharge, whereas Kim et al. [6] noted pain and tenderness in both the breasts. Ko et al. [7] observed tenderness of left breast with volume loss, painful swelling of left lower abdominal wall, and abscess in the left vulva in his patient. In the patient examined by Arslan et al. [8], pain, redness, and deformity of both breasts were observed. Ozcan et al. [2] reported progressive swelling of the right breast in one patient and mastalgia in another patient. Three patients examined by Son et al. [4] complained of palpable lump on the left upper parasternal area (Patient 1), migration of filler (Patient 2), pain, and hardness in the left lower quadrant of the abdomen and breast fistula (Patient 3). In the present study, we also observed many similar symptoms in the patients examined, further strengthening the probable detrimental impact of Aquafilling^®^ injection on the patient’s health.

Intensive inflammation was visible in the examined histological and immunohistochemical samples and could be credited to the ability of T cells to recognize the breast augmentation filler—Aquafilling^®^. Recognition of antigens by T cells is a crucial step in the initiation and regulation of adaptive immune response. T cells having recognized foreign antigens release a large number of chemical factors enabling B cells to create specific antibodies. They activate scavenger cells, including macrophages. The activation process is a result of synergy and completion of specific and unspecific immune response mechanisms. T cells have T cell receptors (TCRs) that enable precise recognition of foreign antigens. Antigens bound by TCR create activation microclusters (MCs). TCR-MC consists not only of TCR receptor with CD3, CD4, or CD8 complex but also of kinase, adaptor protein, and proteins responsible for cytoskeleton alternation. CD3 consists of four different peptide chains that together with TCR are responsible for sending activation signals to Tc lymphocytes (cytotoxic) and Th lymphocytes (helper cells) [11].

In the present study, enhanced expression of T lymphocytes (CD3) and macrophages (CD68), as well as a large immune response area, which in our opinion was observed and can be the result of Aquafilling^®^ recognition by T cells in an unspecific immune mechanism.

Since we examined a small sample group, we could not elucidate other possible mechanisms contributing to the observed variation. However, based on histopathological and immunohistochemical tests, we could surely conclude that the examined patients exhibited aggressive inflammation profiles with long-lasting complications.

In our opinion most likely, we observed that T cells recognized amide bonds of the filler, and hence, antibodies were created by plasma cells against it. Similarly, the presence of macrophages could be related to the removal of the polymer recognized by T cells.

Immunological reaction in the tissues may be associated with the amount of filler injected and the total time elapsed since the injection procedure as observed by extensive inflammatory infiltration and granuloma, fibrous connective tissue partly hyalinized, and the presence of numerous small blood vessels in the histological tissues. However, these results need to be validated in a large sample size.

Intensive chronic inflammation may lead to the development of neoplasia, and therefore, all patients must be informed about this possible risk. As far back as in the year 1863, based on his clinical observations and histopathological research, Rudolf Virchow noted the association between tumor growth and previous lymphocytic infiltration [12]. Later, numerous studies confirmed the dependence between the inflammatory state and neoplasm’s growth. Tumor development in the process of chronic inflammation has been seen in multiple carcinomas including mesothelioma and lymphoma [13–15]. Therefore, choosing an optimal treatment strategy is vital. In the existing literature, no information on how to deal with health issues caused by Aquafilling^®^ injection is available. Jin et al. [16] and Luo et al. [17] presented treatment methods they used in patients when complications caused by breast enlargement with polyacrylamide (PAAG) appeared. Jin et al. [16] suggested an endoscopic or open approach to remove the filler, whereas Luo et al. [17] recommended an open approach. Based on our observations, it occurs that Polish hospitals/clinics use diverse treatment methods in patients after Aquafilling injection, starting from only checking up on a patient and performing ultrasound with elastography, through performing skin incisions, applying and rinsing drains or removing it with liposuction or at last performing vast tissues cleaning during open surgery.

Based on the observations in the present study, it is recommended that every patient who has had injected Aquafilling^®^ (irrespective of procedure time and of visible symptoms being observed) should undergo an ultrasound scan or magnetic resonance imaging (MRI) at first, MRI being more precise. Furthermore, it is advised to remove the filler with the highest precision along with the changed surrounding tissue, through a surgical procedure. Follow-up MRI should be performed 6 months after the surgery. After this time, breast reconstruction using breast implants can be considered.

## Conclusions

Injection of Aquafilling^®^ may trigger an immune response as observed by the heightened inflammatory response in examined tissue samples. Thus, it is highly recommended to perform breast medical imaging such as ultrasound, ultrasound with elastography, or MRI in all patients who had breast enlargement using Aquafilling^®^, even if no visible symptoms or ailments are observed. In patients suffering from inflammation, thorough removal of Aquafilling from all tissues (skin, connective tissue, muscle) that come into contact with the filler, irrespective of visible symptoms or ailments, is highly recommended in order to minimize the complications and to discourage neoplasia process. The influence of time elapsed since the Aquafilling^®^ injection procedure and the amount of the intensity of the immune response of tissue expression require further research.
